# Single-Layer Diverse Chiral Metasurfaces Driven by Generative AI

**DOI:** 10.1021/acs.nanolett.6c04776

**Published:** 2026-10-29

**Authors:** Masanobu Iwanaga, Mihir Kumar Sahoo, Chun-Hao Chiang, Ya-Lun Ho, Keisuke Watanabe

**Affiliations:** † Research Center for Electronic and Optical Materials, 52747 National Institute for Materials Science (NIMS), 1-1 Namiki, Tsukuba 305-0044, Japan; ‡ Research Center for Materials Nanoarchitectonics (MANA), National Institute for Materials Science (NIMS), 1-1 Namiki, Tsukuba 305-0044, Japan

**Keywords:** chiral metasurface, single-layer metasurface, silicon, circular dichroism, generative AI

## Abstract

Optical chirality has been extensively explored in chiral molecules, helical structures, and metasurfaces with bound states in the continuum. Attempts to attain chirality have mainly relied on human intuition and ideas. Here, we show various types of single-layer silicon-nanostructured metasurfaces exhibiting prominent circular dichroism (CD) containing perfect, broadband, and opposite CD responses at short near-infrared wavelengths. The designs were obtained based on a generative AI-assisted structural search for chiral metasurfaces, enabling a series of nonempirical structural searches beyond the boundary of human knowledge and experience. The physical mechanisms yielding the outstanding CD responses are elucidated from detailed insights into subwavelength electromagnetic dynamics in the single-layer metasurfaces. Furthermore, we experimentally demonstrate several metasurfaces. This type of single-layer thin CD metasurface serves as a key element in the development of compact optical-wave-manipulating devices at telecommunication bands.

## Graphical abstract

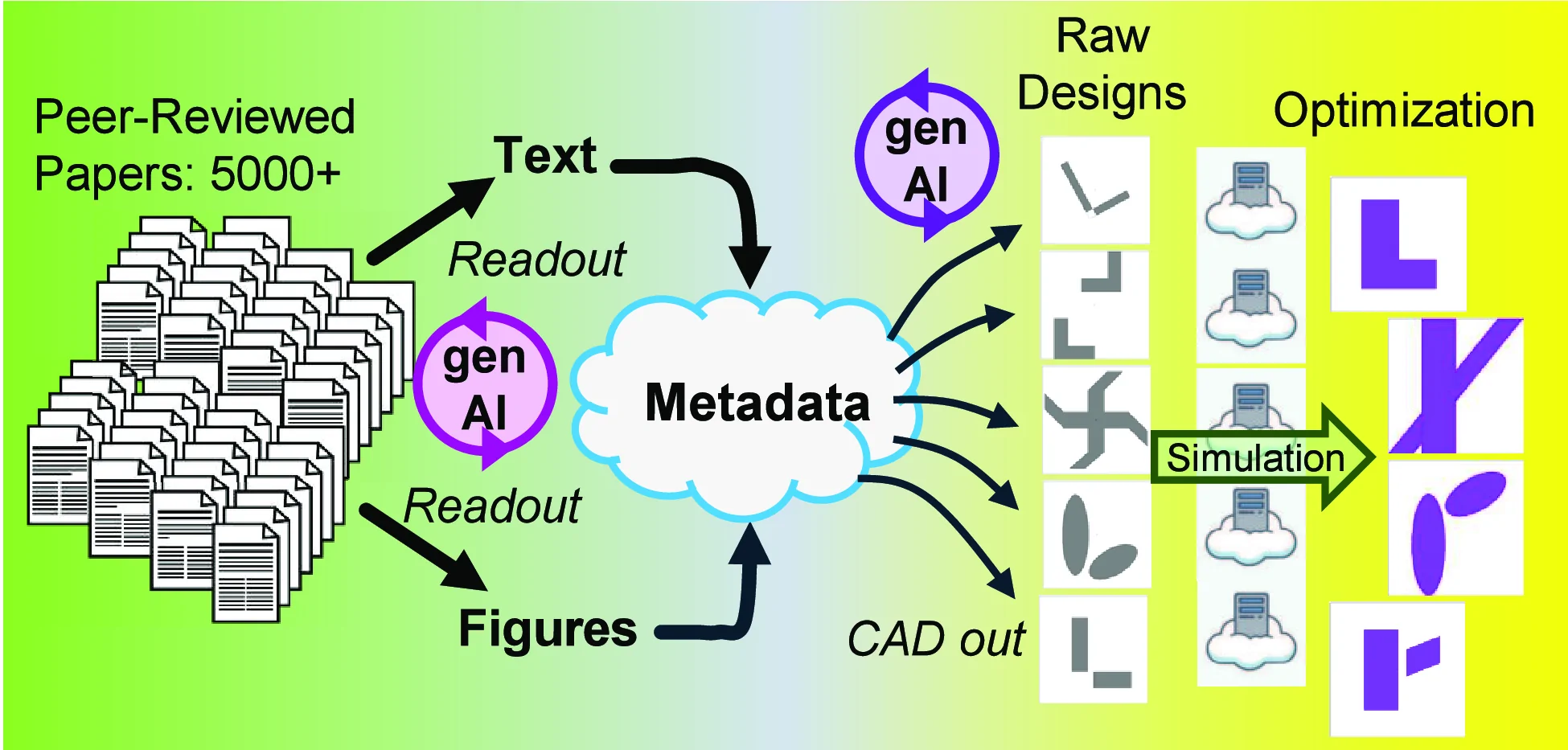

Chiral metasurfaces have been extensively explored and mostly designed by reducing the symmetry of unit cells based on human knowledge and intuition.^1,2^ Some hints were taken from nature, such as chiral molecules^3–5^ and insects.^6^ Other approaches were based on resonance-enhanced chiral responses at optical wavelengths.^7–18^ In terms of lowering symmetry, bound states in the continuum (BIC)^7,9,10,12,14,16–18^ and diffractions^12,13^ were incorporated to access chiral responses. Despite the great efforts to produce chiral metasurfaces, the explored portions were still limited to a part of the diverse possibilities. From a practical viewpoint, feasibility in production, robustness in operation, and compact configurations in use are generally preferred, thereby discouraging elaborate stacked-layer structures,^8,13^ single-frequency (or very narrow band) operation,^7,9,10,12,14,16–18^ and complicated detection setup.^12,13^ Thus, there is demand for further exploration of the possibility of functional chiral metasurfaces that have not yet been discovered. Such chiral metasurfaces are likely to expediate detection of chiral molecules in practical configurations.^19–21^

A new approach to exploit generative AI (gAI) was recently introduced for nanophotonics structures,^22^ which first started to prepare metadata using a gAI (Amazon Titan Text G1) based on nanophotonics literature exceeding 5000 published papers, second output candidates for chiral metasurfaces through a question-and-answer manner with human language, and finally translated the outputs to computer-aided design (CAD) files with help of another gAI (Anthropic Claude 3.5). The entire procedure is schematically illustrated in Figure 1A. The preparation of the specialized metadata is crucial^22^ because current commercial AIs output hallucinations to scientific questions. Going through the procedure on a cloud service,^23^ the designs were output based purely on scientific metadata extracted from published scientific papers, thereby being free from personal intuition and/or inspiration. As a concrete implementation of this exploration system, we explored single-layer chiral metasurfaces working at telecom wavelengths from 1300 to 1600 nm, because chiral metasurfaces are a more complicated subject than subwavelength polarizers and waveplates that can be designed, based on a physical understanding of the working principles.^24–27^

**Figure 1:**
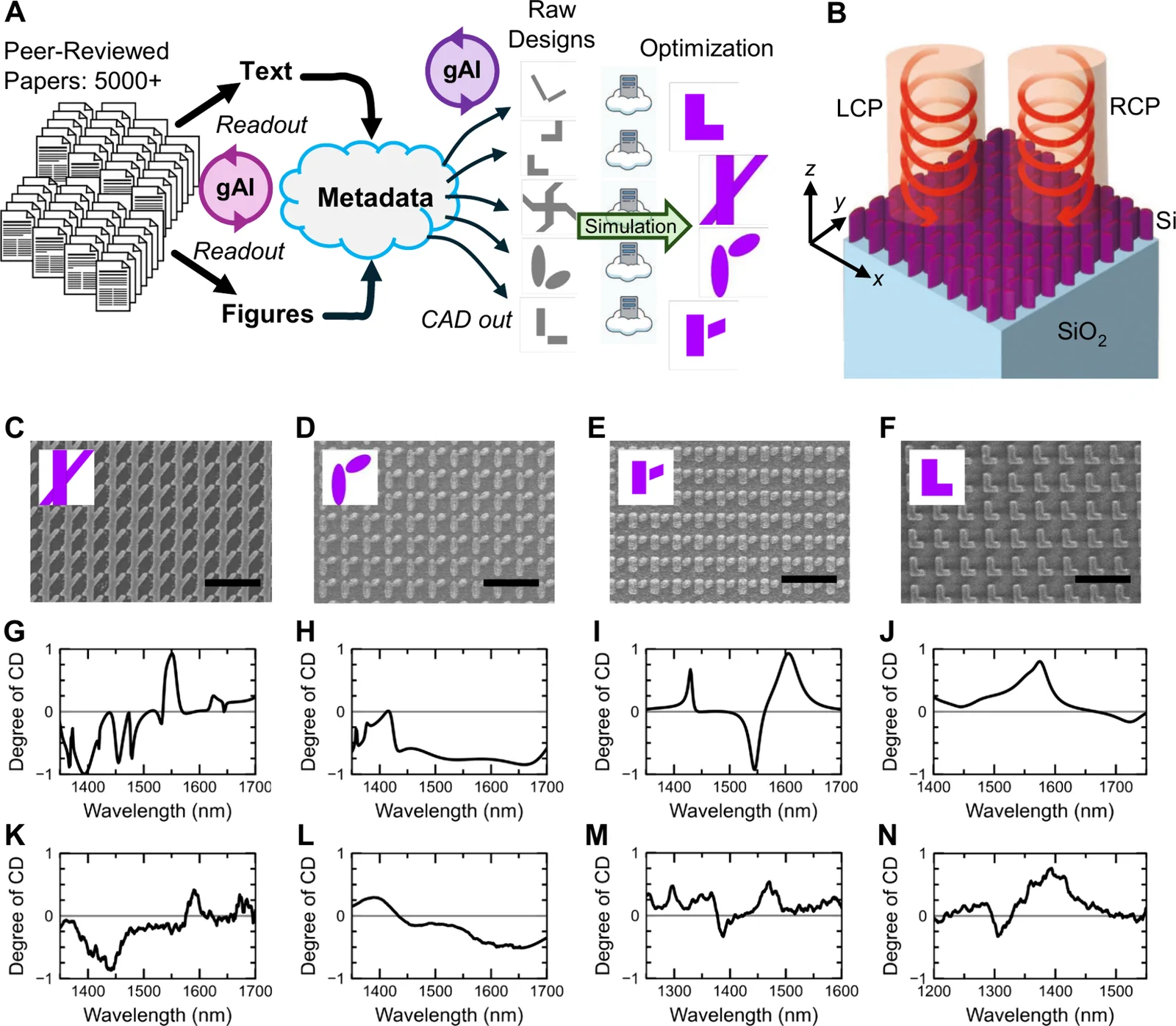
(A) Schematic of the metadata-based design output by generative AI (gAI) to find chiral metasurfaces. (B) Illustration of the optical configuration for circular dichroism (CD) measurement. (C–F) Scanning electron microscopy images of the nanofabricated chiral metasurfaces. Scale bars commonly indicate 2 μm. Insets show the designs of unit cells, representing Si with purple. (G–J) Simulated CD spectra, corresponding to the designs for panels C–F, respectively. The degree of CD is defined by eq 1. (K–N) Measured CD spectra, corresponding to the metasurfaces in panels C–F, respectively.

Searching single-layer silicon (Si) metasurfaces on quartz substrates, we obtained a large number of candidates for metasurfaces exhibiting prominent circular dichroism (CD) effects; concretely, approximately 50 candidates were output, though the number was limited not by the gAI but by us, simply because we cannot handle numerous candidates in reality. The candidates were numerically examined by implementing a full-wave simulation package, Meep,^28,29^ for the CAD files and extended on the same cloud service because any gAI does not have sufficient capability now of outputting precise electromagnetic (EM) quantities regarding metasurfaces. In the simulations, some nanostructures were changed in 10 nm steps in position in the unit cell, and refined structural tuning enabled us to obtain several optimized good designs exhibiting prominent CD effects; furthermore, the thickness of the metasurface was changed in 100 nm steps. For each candidate output by the gAI, we typically conducted 100-case structural tuning, including the thickness.

We briefly compare the gAI-based search to existing techniques, such as inverse design.^30–32^ The former provides various candidates at first to meet our question, e.g., “Can you output 10 good chiral metasurfaces?”, while the latter tries to determine an optical metasurface under a constraint, “Find an optimal CD structure with starting a given structure.” We can further receive other candidates by modifying the question for the gAI-based platform. Thus, the former makes us access diverse candidates in a shorter time than the latter, being more productive. After finding diverse candidates in the former, we proceed to further structural tuning and can select the most preferable metasurface(s), whereas in the latter, we have only one metasurface in a sequence of search. We note that a topological optimization technique^20^ is conducted similarly to the inverse design.

Although the promising designs for single-layer chiral metasurfaces were obtained through the gAI-based procedure (Figure 1A),^22^ the elucidation of the mechanism to yield prominent CD and the experimental demonstration have not been attained, which are addressed in this Letter. We emphasize that, when gAI-based designs are obtained for scientific purposes, the gAI does not clarify the underlying physical, chemical, and biological mechanisms; therefore, we further need to conduct scientific approaches to the gAI-based outputs and to clarify the mechanism and limitation. This will be a general standard when gAIs for science are used.

In Figure 1B, the optical configuration to demonstrate the CD effect experimentally is illustrated, thereby including the *xyz* axes. The incidence of left- and right-handed circular polarizations (LCP and RCP, respectively) normally sheds light on the single-layer Si metasurfaces on quartz substrates. Using transmittance under LCP and RCP (*T*_LCP_ and *T*_RCP_, respectively), we here define the degree of CD, σ_CD_, as follows:

1 $$\sigma_{\mathrm{CD}}=\frac{T_{\mathrm{RCP}}-T_{\mathrm{LCP}}}{T_{\mathrm{RCP}}+T_{\mathrm{LCP}}}$$

Index σ_CD_ is an indicator of the CD effect, evaluating the chiral metasurfaces quantitatively.

Figure 1C–F shows top-view scanning electron microscopy (SEM) images of gAI-driven single-layer Si metasurfaces realized in experiment; scale bars indicate 2 μm in common. Insets represent the designs obtained through the gAI-based procedure (Figure 1A). To find chiral metasurfaces working efficiently in a wavelength range around 1500 nm, the dimensions of square unit cells in Figure 1C–E were determined to be 800 nm × 800 nm and that in Figure 1F to be 900 nm × 900 nm. The height of Si nanostructures in the unitcell was designed to be originally 800, 900, 1000, and 500 nm in Figure 1C–F, respectively. To obtain better correspondence of the simulation to the measurement, Si-on-quartz (SOQ) thickness was experimentally determined by measuring transmittance of SOQ wafers and reproducing it by simulation (Supporting Information, Figure S1).

The metasurfaces in Figure 1C–F correspond to numerically calculated and measured results in Figure 1G–J and 1K–N, respectively. The measurement details are noted in section S1. The nanofabrication was conducted through electron-beam lithography and deep reactive ion etching (RIE) for the SOQ layers selectively (section S2). The simulated results indicating perfect, broadband, and opposite CD effects are basically reproduced in the measured results. In particular, almost perfect CD effects are realized in the experiment for the crossbar and L-shape metasurfaces (Figure 1K,N). We note that, in the measured CD spectra, there are some CD reductions (Figure 1L,M) and a working-wavelength difference (Figure 1N) from the calculated ones. We attribute these to structural deviations from the designs; for example, the sizes of each nanoblock and nanorod tended to be reduced, and sharp corners in the designs were not ideally realized in the nanofabrication, irrespective of our frequent trials.

The transmittance spectra and EM-field distributions were computed by executing a rigorous coupled-wave analysis (RCWA) code combined with a scattering matrix algorithm.^33,34^ The self-developed RCWA code was executed on supercomputers in a multiparallel implementation manner. The permittivity of Si was taken from literature,^35^ and that of SiO_2_ and air was set to a representative value of 2.1316 and 1.00054, respectively.

Underlying mechanisms yielding the CD effects in the metasurfaces are described in the following. Figure 2A depicts 3D-view unit cell of metasurface featuring the crossbar structure (Figure 1C), together with *xyz* axes and incident wavevector **k**_in_ (red arrow). The incidence propagates along the −*z* direction, being normal to the metasurface. The unit cell is 800 nm square in the *xy* plane, and the height is set to 780 nm, in accordance with the experimental SOQ wafer (Figure S1).

**Figure 2:**
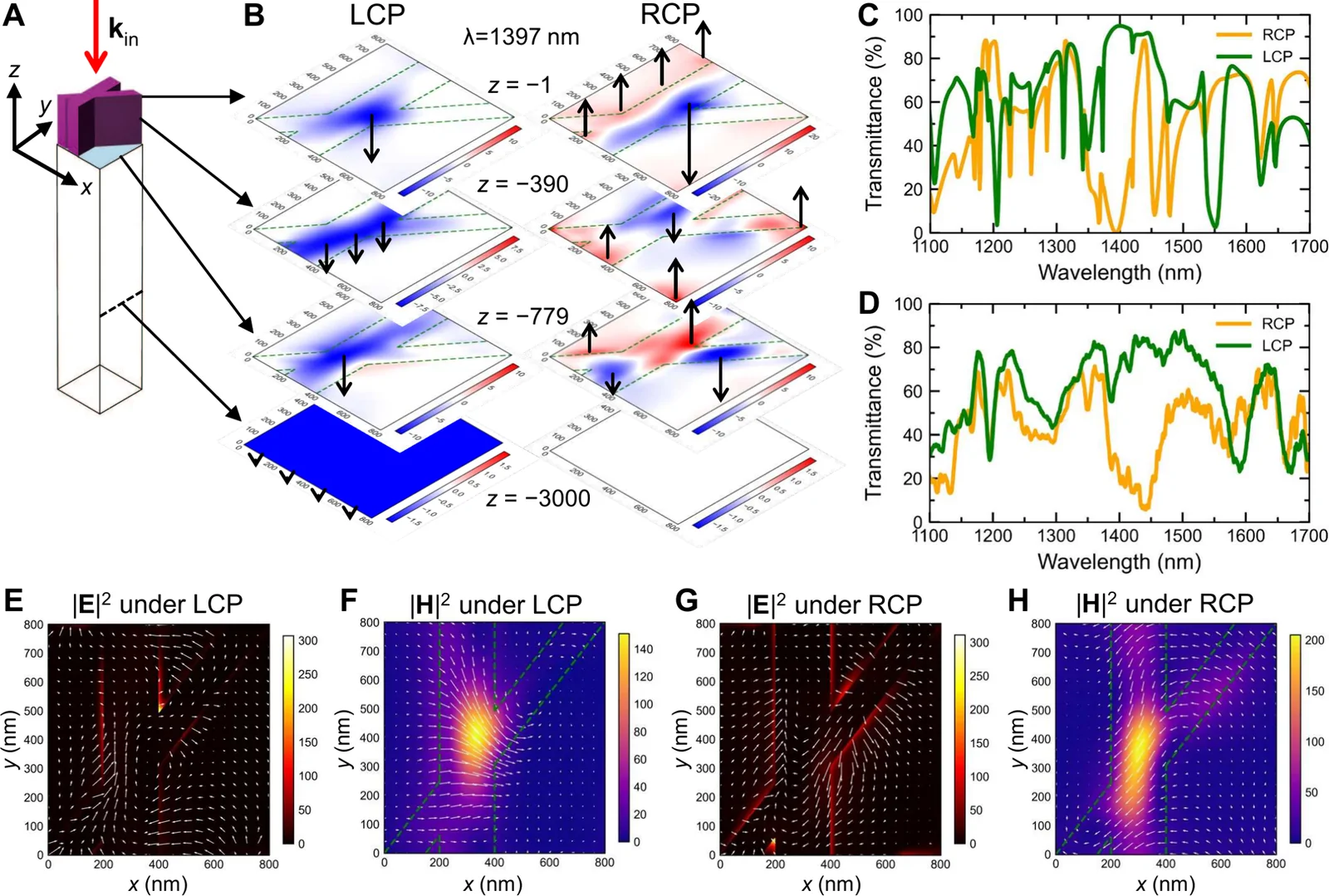
(A) 3D-view unit cell of the crossbar structure. (B) *xy* section views of *S*_*z*_ components at *z* values of −1, −390, −779, and −3000 nm for λ = 1397 nm. (C and D) Computed and measured transmittance spectra under RCP (orange) and LCP (green), respectively. (E–H) Electric- and magnetic-field intensity, |**E**|^2^ and |**H**|^2^, under LCP and RCP, respectively, corresponding to the *xy* section at a *z* of −779 nm in panel B.

At incident wavelength λ = 1397 nm, where σ_CD_ ≈ −1 (Figure 1G), we examine the mechanism to yield the almost perfect CD effect, which is associated high-contrast transmittance. Figure 2B shows Poynting-vector component *S*_*z*_ in *xy* sections; each *z* value is shown, representing the top, middle, and bottom of the crossbar structure at *z* values of −1, −390, and −779 nm, respectively. Also, a set of far-field transmission sections is shown at a *z* of −3000 nm. The position of the crossbar structure is shown with dashed green lines. The *S*_*z*_ component is displayed in blue–white–red color representation, where blue and red denote negative and positive values, respectively. Representative *S*_*z*_ fluxes are indicated by arrows. We note that the incidence was set to be |**E**| = 1 and |**H**| = 1; consequently, |**S**| = 1. Under LCP, the EM wave is effectively transmitted in the crossbar structure; in other words, the crossbar functions as an efficient circular-polarization selective waveguide.

Panels C and D of Figure 2 show numerically calculated and measured transmittance spectra, respectively, under LCP (green) and RCP (orange). The highest contrast of the calculated transmittance (*T*_LCP_ > 93.5%, and *T*_RCP_ < 0.6%) appears at 1391.1–1395.8 nm (Figure 2C), whose mechanism was examined in detail in Figure 2B. Accordingly, a high contrast of *T*_LCP_ > 83.1% and *T*_RCP_ < 6.7% was observed in the range of 1439–1442 nm in the measurement (Figure 2D). These transmittance responses are the origin of an almost perfect CD effect (i.e., σ_CD_ ≈ −1) in panels G and K of Figure 1. We infer that the deviation of the working wavelengths and transmittance came from the structural deviation of the actual sample from the design (Figure 1C).

The resonant electric- and magnetic-field intensities, |**E**|^2^ and |**H**|^2^, on an *xy* section at a *z* of −779 nm under LCP are shown in panels E and F, respectively, of Figure 2. The |**E**|^2^ and |**H**|^2^ distributions are displayed in hot and plasma color representations, respectively, together with scale bars. The *x* and *y* components of electric- and magnetic-field vectors are also presented with white arrows. A prominent hot spot of |**E**|^2^ > 300 is observed at a joint of the crossbar regarding the |**E**|^2^ distribution (Figure 2E), which is mostly composed of a near-field *E*_*z*_ component and does not contribute to EM-wave propagation, that is, the *S*_*z*_ component. Regarding the |**H**|^2^ distribution (Figure 2F), strong localization occurs inside the Si crossbar structure, which is associated with the *xy*-vector fields and contributes to the predominant EM-wave propagation (blue) that manifest itself at the left section of *z* = −779 nm in Figure 2B.

The |**E**|^2^ and |**H**|^2^ distributions under RCP are shown in panels G and H, respectively, of Figure 2. They are qualitatively similar to those in panels E and F of Figure 2, in terms of hot |**E**|^2^ spots at the rim of the crossbar structure and localization of |**H**|^2^ inside it. Thus, the mechanism of the prominent CD is indefinite only from these EM-field distributions. In contrast, the *S*_*z*_ fluxes (Figure 2B) offer comprehensive EM dynamics in the unit cell. This kind of approach is valid for nontrivial and complicated subwavelength EM dynamics.^36,37^

Figure 3A shows a 3D view of the unit cell of the elliptical pair metasurface. The incidence propagates along the −*z* direction, shedding normally on the metasurface placed in the *xy* plane. The unit cell is 800 nm square in the *xy* plane, and the height is set to 878 nm, which was determined based on the measured transmittance of the SOQ wafer (Figure S1).

**Figure 3:**
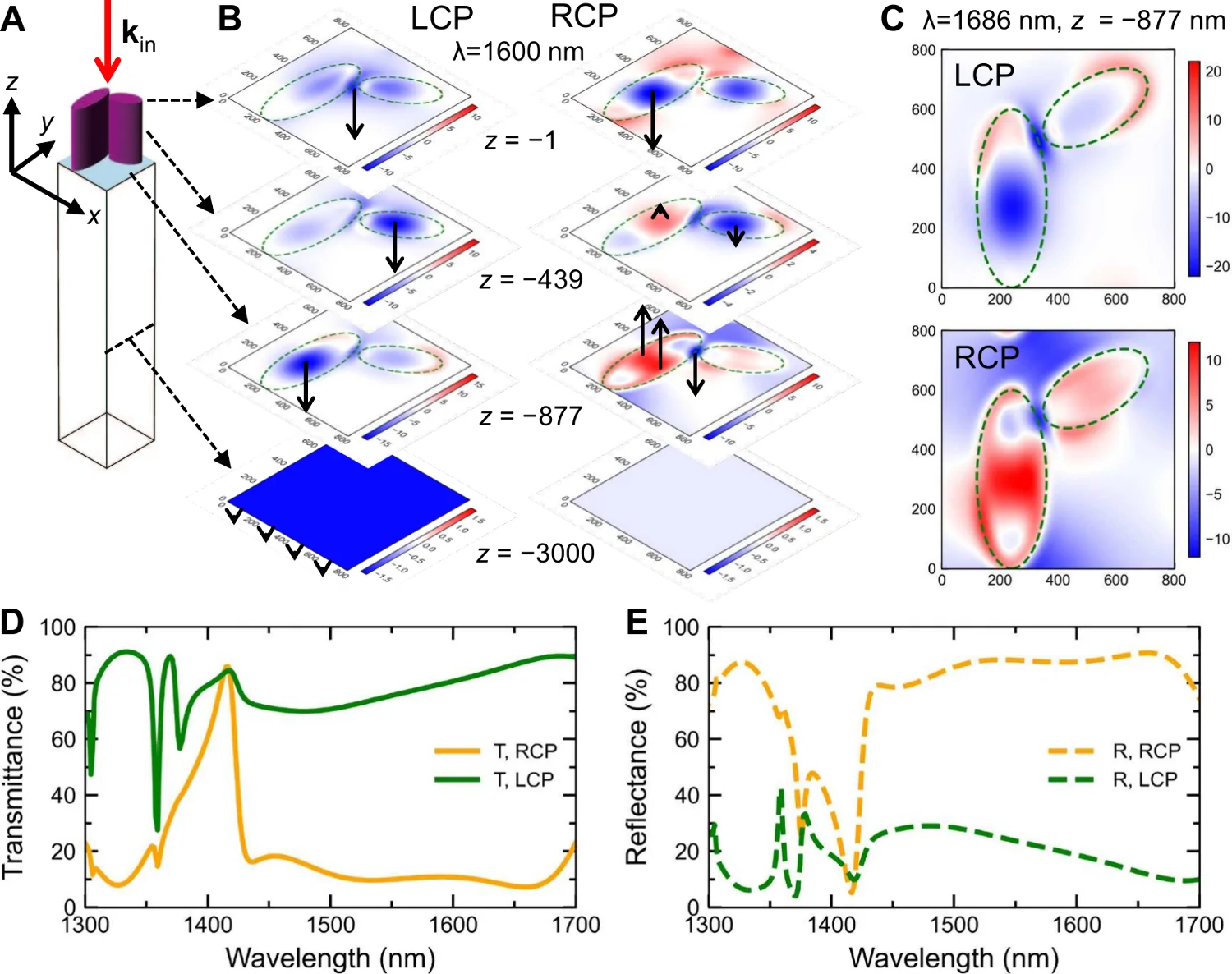
(A) 3D illustration of the unit cell of the elliptical pair. (B) Poynting *S*_*z*_ components at λ = 1600 nm under LCP (left column) and RCP (right column), shown in *xy* section views, whose *z* positions in nanometers are indicated by dashed-line arrows from panel A. Red and blue denote +*z* and −*z* components, respectively. Dashed green curves show the positions of the elliptical pair. Representative *S*_*z*_ components are indicated by arrows. (C) *S*_*z*_ components at λ = 1686 nm under LCP (upper) and RCP (lower), shown at *z* = −877 nm. (D and E) Computed transmittance and reflectance spectra, respectively, under RCP (orange) and LCP (green).

In Figure 3B, *S*_*z*_ distributions at λ = 1600 nm in the middle of the broad CD band (Figure 1H) are shown. The *xy* sections are taken at *z* values of −1, −439, −877, and −3000 nm. Inside the unit cell, the *S*_*z*_ fluxes under LCP are distinct from those under RCP. Under LCP, EM wave effectively transmits in the two elliptical Si nanorods; in particular, it goes forward in the smaller rod at the middle (*z* = −439 nm) as represented with blue, while it transmits in the larger rod at the bottom (*z* = −877 nm). In contrast, under RCP, forward (blue) and backward (red) *S*_*z*_ fluxes coexist in the elliptical rods. At the bottom of *z* = −877 nm, the backward component becomes predominant; consequently, the far-field transmittance is significantly smaller than that under LCP at *z* = −3000 nm. The EM dynamics is true for a longer wavelength of λ = 1686 nm, as shown in Figure 3C.

Simulated transmittance and reflectance spectra of the elliptical pair metasurface are shown in panels D and E, respectively, of Figure 3, where the green and orange curves represent the spectra under LCP and RCP, respectively. The transmittance and reflectance are complementary under each polarization; thus, they are hardly associated with optical loss. Thus, the metasurface exhibits broadband CD effects in both transmittance and reflectance. We also note that the broadband CD responses are maintained for incident angles up to 12°, which is one of the widest responses (or flat CD continuum) reported to date.

The broadband CD effect observed in experiments (Figure 1L) though the wavelength range of σ_CD_ ∼ 0.5 was limited to a range of 1580–1680 nm, being narrower than the simulation. This was directly affected by an increase in the measured *T*_RCP_ in the range of 1450–1580 nm (Figure S3A). From numerical *S*_*z*_ flow at 1450 nm in the designed structure, strong suppression of *T*_RCP_ turns out to occur at the rim of the larger elliptical rod (Figure S4); however, the rod was unexpectedly shrunk by approximately 20 nm in the nanofabrication, compared to the design, and the rim did not work.

The broadband CD response of the elliptical pair metasurface originates from the circular polarization-dependent transmission mode in the Si rod pair (Figure 3B). After the search and experiment, we noticed references addressing broadband CD responses.^38–40^ A design mimicked the concept of quarter waveplates and comprised four rectangular TiO_2_ nanorods in the unit cell, which was experimentally validated to work at λ = 520–600 nm.^38^ Another design comprised two rectangular Si nanorods contacting at a sharp corner, which was shown to work at λ = 1520–1600 nm only by simulation;^39^ from an experimental viewpoint, such sharp corners are difficult to realize. The other design comprised a periodic array of straight Si waveguide with protrusions;^40^ by adjusting the shapes of protrusions, a large CD effect was experimentally observed at λ = 1430–1600 nm, whereas the mechanism of the CD effect is not explicitly revealed, though it was attributed to multipoles. Thus, the designs for broadband CD effects are quite different from each other. We newly introduce a simple design in Figure 3.

Elliptical pair structures of Si_3_N_4_ of a single layer were numerically studied.^16^ However, any broadband CD effect was not found, and a narrowband CD response on BIC was addressed under oblique incidence. Thus, it requires substantial effort to find the broadband CD effect in Figure 3, even when elliptical pair structures are explored.

Figure 4A illustrates 3D unit cell of oblique-block metasurface in a manner similar to that depicted in Figure 3A. The unit cell is 800 nm square in the *xy* plane, and the height is set to 975 nm, which was the thickness of the SOQ layer (Figure S1).

**Figure 4:**
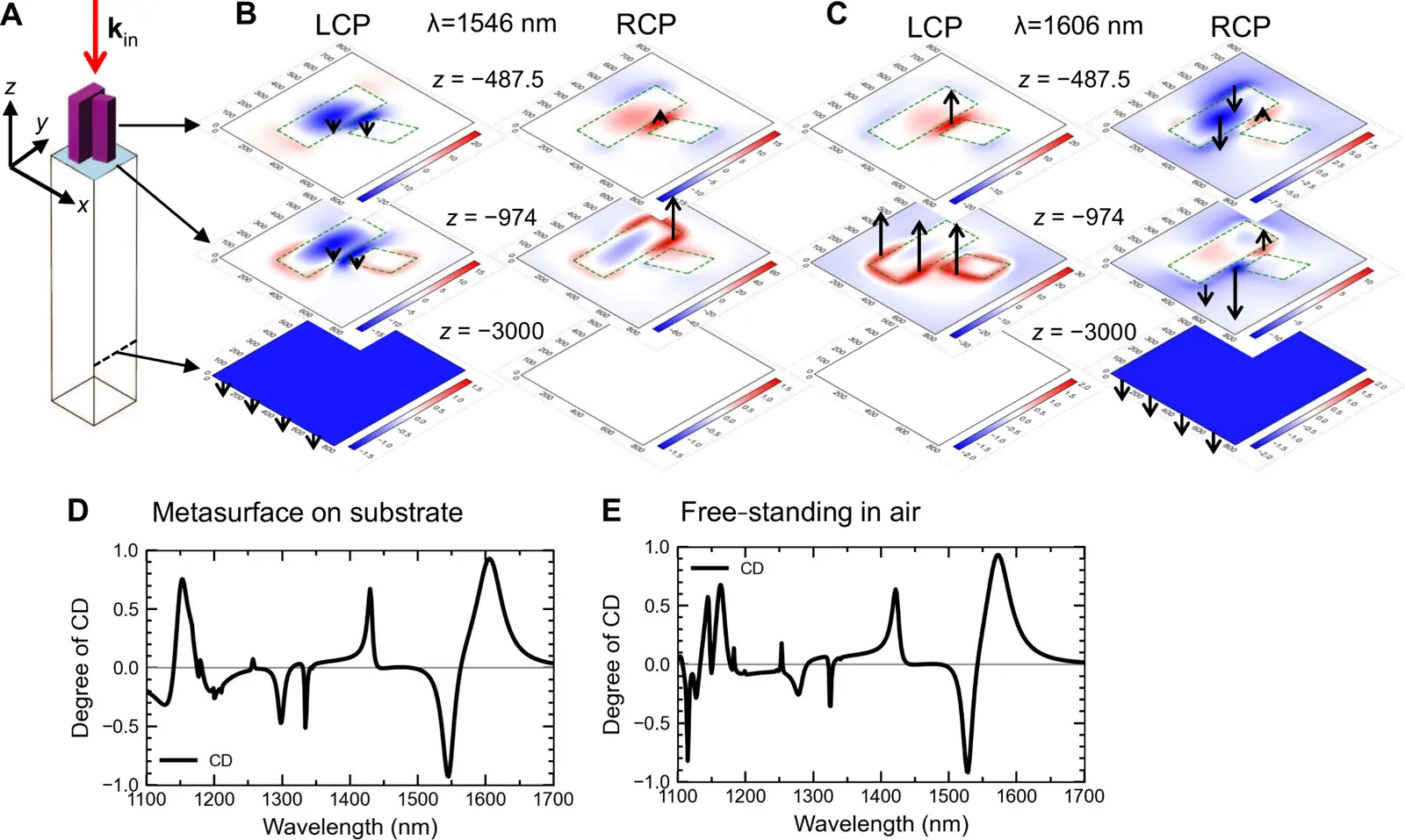
(A) 3D illustration of unit cell of a pair of rectangular and oblique blocks. (B and C) 3D-slice views of *S*_*z*_ components contributing to σ_CD_ = −1 and 1 at λ = 1546 and 1606 nm, respectively. The *xy* slices are taken at *z* values of −487.5, −974, and −3000 nm. Green dashed lines indicate the position of oblique Si blocks. (D and E) Computed CD spectra of the metasurfaces on the substrate and free-standing in air, respectively.

In panels B and C of Figure 4, *S*_*z*_ components are presented at λ values of 1546 and 1606 nm, which correspond to σ_CD_ values of −1 and 1, respectively. Under LCP at λ = 1546 nm (Figure 4B), forward *S*_*z*_ flux mainly localizes in the larger block and the nanogap between the blocks at the middle and bottom of blocks (*z* = −487.5 and – 974 nm, respectively), whereas under RCP, backward flux is predominant and appears around the blocks. As a result, transmittance is large only under LCP. At λ = 1606 nm (Figure 4C), the EM dynamics changes significantly; under LCP, backward flux is dominant, while under RCP, forward flux localizes inside and around the larger block at the middle and concentrates in the nanogap at the bottom. Consequently, large transmittance occurs only under RCP. In the oblique blocks, the nanogap plays a key role in the large transmittance, which is considered to affect experimental results (Figure S3B and Figure 1M).

In the design of Figure 4, the nanogap width was 50 nm. The positions of the smaller block to the larger one also affect the CD effects (Figure S5). We show the optimal position in Figure 4 or inset of Figure 1E. When increasing the nanogap width to 100 nm, the CD effects tend to be reduced more (Figure S6). These evaluations were implemented in the structural tuning step, as shown in Figure 1A.

To determine whether the prominent CD effect is intrinsic or extrinsic, we computed CD spectra of the oblique-block metasurfaces with and without a substrate, as shown in panels D and E, respectively, of Figure 4 because CD effects were often reported under low-symmetry optical configurations, such as oblique incidence.^15,41^ In the diffraction-free subwavelength range longer than 1200 nm (Figures S7–S9) and 1450 nm (Figure S10), prominent peaks of the CD spectra under normal incidence are similar in the two configurations; therefore, we can declare that the substrate effect is subsidiary and that the CD effect intrinsically comes from the metasurface itself, even under a symmetric optical configuration. This property holds true for all the metasurfaces addressed in this Letter.

Regarding the L-shaped metasurface, the subwavelength EM dynamics in the unit cell is shown in Figure S11. Forward and backward *S*_*z*_ fluxes are more competitive than those in other metasurfaces (Figures 2–4). Nevertheless, the forward *S*_*z*_ is predominant under RCP, resulting in high transmittance and a large positive σ_CD_ (Figure 1J,N). This behavior was observed in measured transmittance spectra (Figure S3C), resulting in the large CD effect.

In conclusion, we have shown diverse chiral metasurfaces that are made of single-layer Si nanostructures, which feature outstanding CD responses at telecom wavelengths. The designs were obtained through gAI-based exploration, and insight into the CD effects was made explicit by visualizing the subwavelength EM dynamics. The revealed dynamics has not been predicted, to the best of our knowledge, exhibiting diversity. Even when structures are similar at a glance (e.g., Figure 1D,E), the underlying EM dynamics is quite different and consequently enables unique CD effects, such as perfect, broadband, and opposite CDs. The designs were realized fairly well in the experiment, which strongly supports the practical potential for compact CD devices with subwavelength thickness at telecom wavelengths.

## Supplementary Material



## Notes

Optical measurement and determination of SOQ thickness based on transmission spectra (Figure S1), nanofabrication procedure, including optimized deep Si RIE (Figure S2), measured transmittance spectra (Figure S3), *S*_*z*_ distributions of the elliptical pair metasurface at 1450 nm (Figure S4), structural tuning of the oblique block unit (Figures S5 and S6), examinations of the substrate effect on CD (Figures S7–S10), and CD-effect-yielding *S*_*z*_ distributions of the L-shaped metasurface (Figure S11) (PDF)

## ¶

International Center for Young Scientists (ICYS), National Institute for Materials Science (NIMS), 1-1 Namiki, Tsukuba 305-0044, Japan
