# Supplementary material for: Single-Layer Diverse Chiral Metasurfaces Driven by Generative AI
Source: Nano Lett. 2026 Oct 29;26(44):15483–9. doi: 10.1021/acs.nanolett.6c04776 (PMC13761687; doi:10.1021/acs.nanolett.6c04776)
Supplement: Supplementary file 1 [file nl6c04776_si_001.pdf]

Supporting Information: Single-Layer Diverse Chiral Metasurfaces Driven by Generative AI

Masanobu Iwanaga,[*,†] Mihir Kumar Sahoo,[†] Chun-Hao Chiang,[†,¶] Ya-Lun Ho,[†] and Keisuke Watanabe[‡]

†*Research Center for Electronic and Optical Materials, National Institute for Materials Science (NIMS), 1-1 Namiki, Tsukuba 305-0044, Japan*

‡*Research Center for Materials Nanoarchitectonics (MANA), National Institute for Materials Science (NIMS), 1-1 Namiki, Tsukuba 305-0044, Japan*

¶*Current address: International Center for Young Scientists (ICYS), National Institute for Materials Science (NIMS), 1-1 Namiki, Tsukuba 305-0044, Japan*

E-mail: iwanaga.masanobu@nims.go.jp

Note: In this Supporting Information, the abbreviations defined in the text are used without definitions.

S1. Experimental Determination of SOQ Thickness

Measured transmittance (T) spectra of flat SOQ substrates were reproduced by computing T spectra for various thickness,[S1] as shown in Figure S1. Permittivity of crystalline Si was taken from literature.[S2] The nominal thickness was determined precisely, i.e., in 2 nm precision. The identified thickness of SOQ was used in the text for the computations for T

and CD spectra.

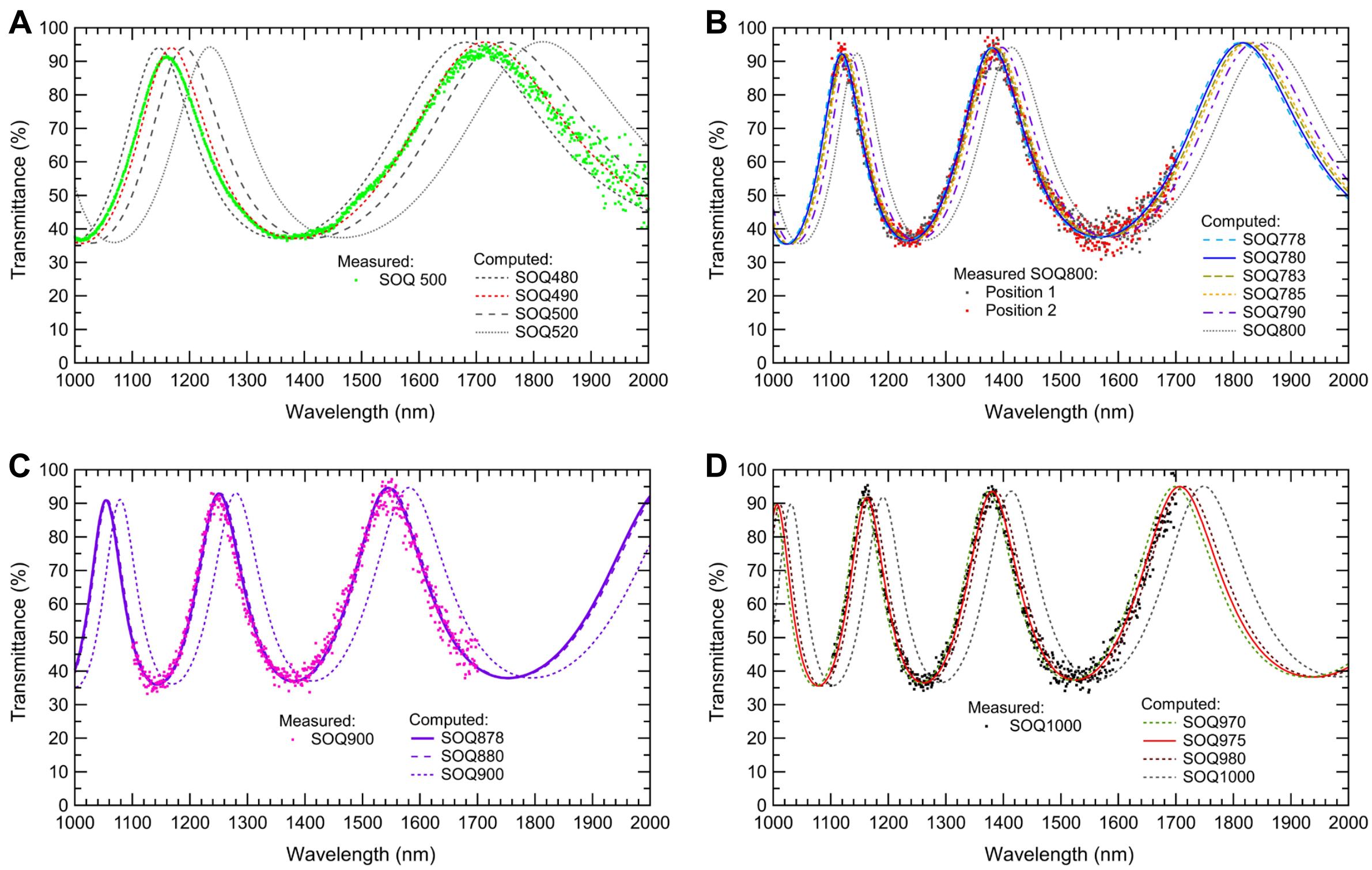

Figure S1: Measured T spectra of flat SOQ substrates versus computed T spectra. (A)–(D) SOQ thickness 500, 800, 900, and 1000 nm was initially assumed, respectively. It was determined to be 490, 780, 878, and 975 nm, respectively, from the best-fitting computed spectra.

The T measurement was conducted in a spectrometer (V-7200, JASCO, Tokyo, Japan). The CD measurements were conducted under circular-polarized incidence, which was transduced from linearly polarized light in the spectrometer using an achromatic quarter waveplate (AQWP05M-1600, Thorlabs Inc., Newton, NJ) in the near-infrared range from 1100 to 1700 nm. The measured CD spectra in Figure 1K–N were smoothed using the Savitzky-Golay algorithm. The incident circular polarizations were verified by measuring the polarization distributions in the plane perpendicular to the incident wavevector.

S2. Si Nanofabrication

In addition to commercially manufactured SOQ wafers with Si-layer thicknesses of 500, 900, and 1000 nm, SOQ wafers with thicknesses of 700 and 800 nm were prepared through the RIE plasma etching process (RIE-200NL, Samco Inc., Kyoto, Japan) from the 900-nm thick wafers. The CD metasurfaces were fabricated through electron-beam (EB) lithography on the SOQ wafers: EB drawing was conducted under a 100 keV acceleration beam (ELS-BODEN, Elionix Inc., Tokyo, Japan) using a positive-type EB resist (FEP-171, FUJIFILM Corp., Yokohama, Japan); going through subsequent development, Si deep RIE was conducted in a BOSCH process instrument (MUC-21 ASE-SRE, Sumitomo Precision Inc., Hyogo, Japan) using the resist mask; finally, the resist remained on the Si nanostructures was removed using an aqua plasma cleaner (AQ-500, Samco Inc.).

Figure S2 shows normal etching of Si layer in an SOQ substrate, conducted through deep RIE. EB-resist patterns were transcribed to the Si layers of SOQ substrates. The etching direction was from top to bottom in Figure S2.

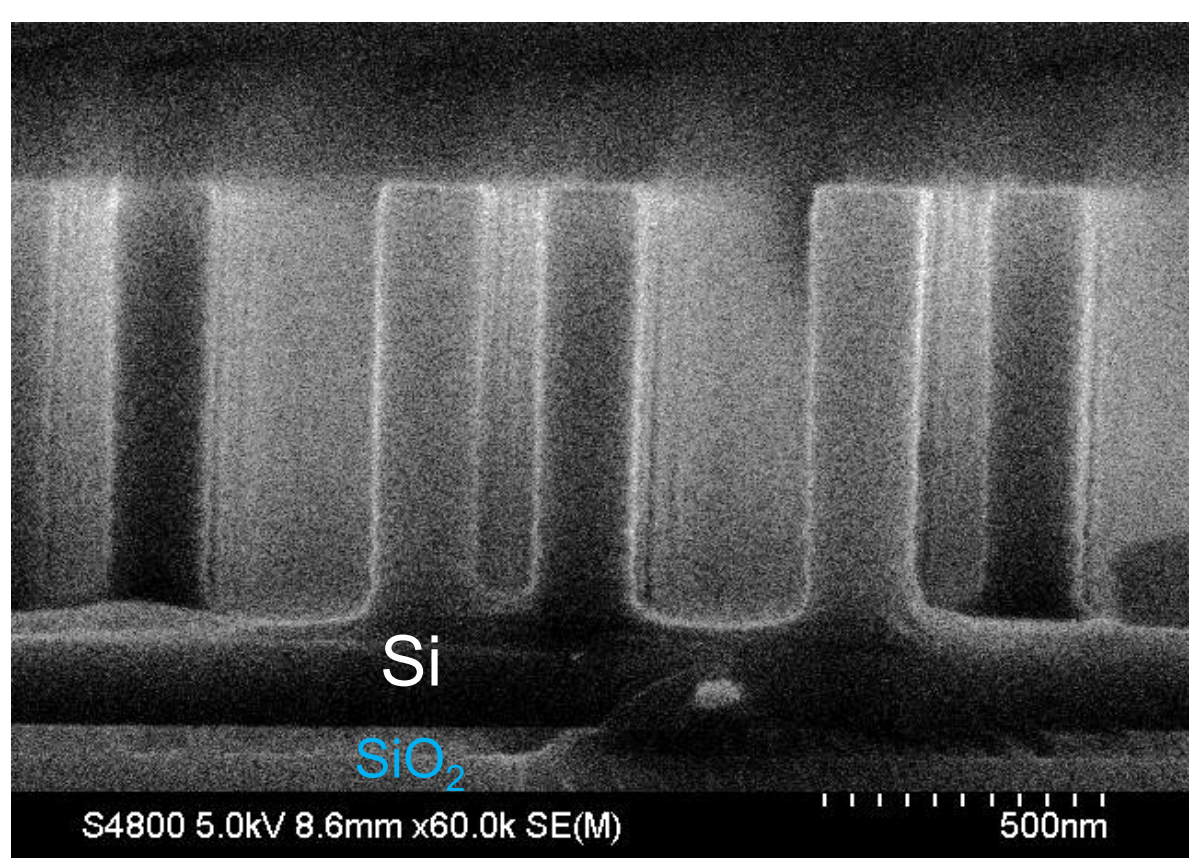

Figure S2: Section-view SEM image after deep Si RIE. Normal etching of Si was conducted on an EB-resist-patterned SOQ substrate; the etching direction was from top to bottom.

S3. Measured T Spectra under Circular Polarizations

Figure S3 shows measured T spectra of three-type metasurfaces: (A) elliptical pair, (B) oblique block, and (C) L shape. The T spectra yield the CD spectra in Figure 1L–N by using eq 1 in the text.

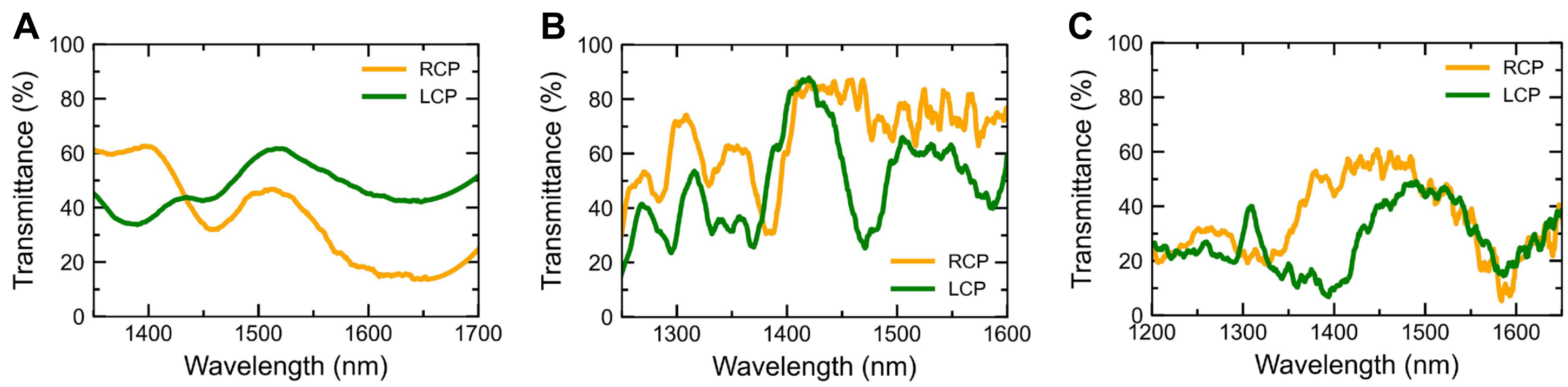

Figure S3: Measured T spectra of the single-layer metasurfaces made of unit structures: (A) elliptical pair, (B) oblique block, and (C) L shape.

S4. S_z Distributions of Elliptical Pair Metasurface

Figure S4 displays numerically calculated S_z distributions at 1450 nm; the z positions of the xy sections are labeled on each panel. Upper raw corresponds to LCP, and lower one to RCP. Color presentation is the same as that in Figure 3B. Strong backward S_z flux appears at the rim of the larger rod under RCP at $z = -877$ nm, playing a major role to suppress T_{RCP}.

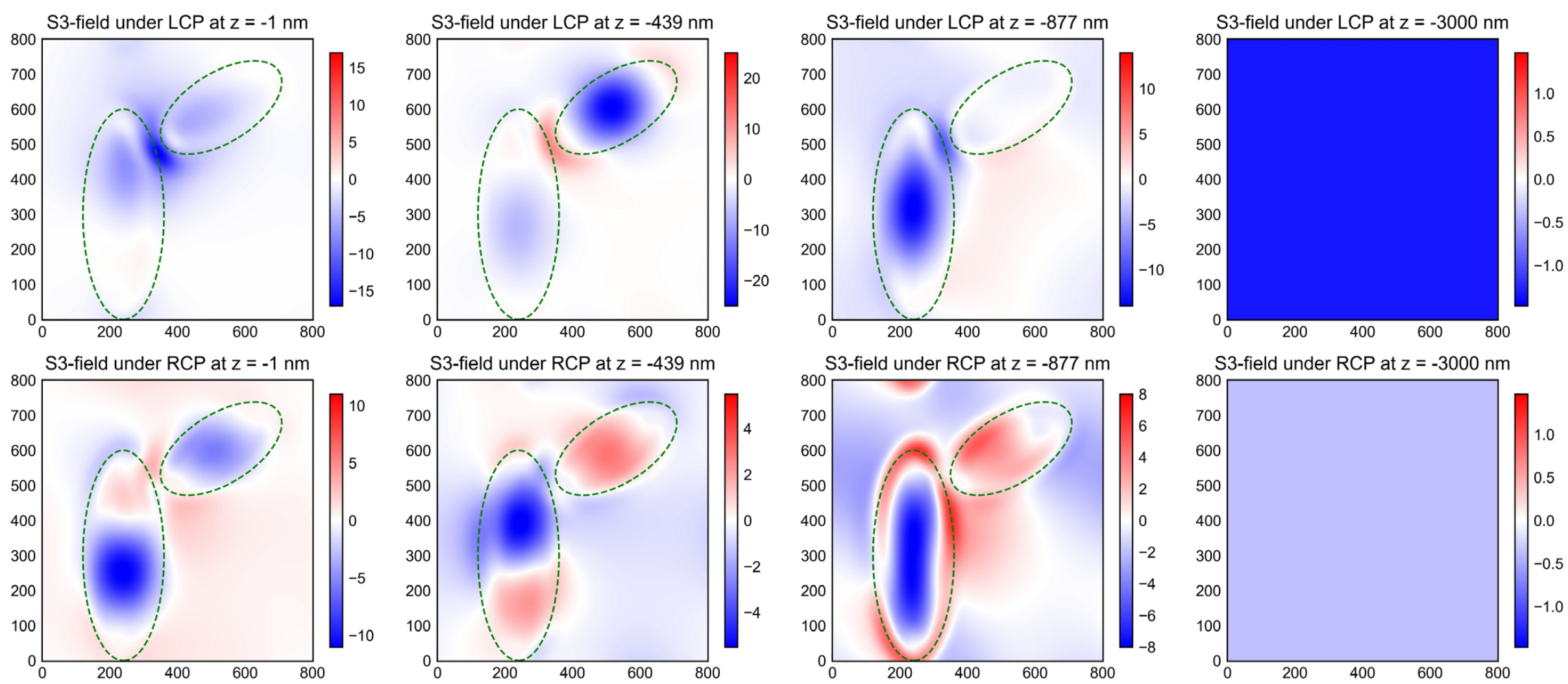

Figure S4: Elliptical pair metasurface: S_z distributions at xy sections at 1450 nm. The z positions are labeled on each panel. We note that S3 is identical to S_z in this figure.

S5. Structural Tuning of The Oblique Block Unit

Figure S5,S6 shows a series of structural tuning results regarding the metasurfaces made of oblique block units. The unit structures deviate from the optimum structure in Figure 1E, showing reduced CD spectra in comparison with Figure 1I. We note that the spectra in Figure S5,S6 were computed by a finite-difference time-domain method using a package Meep.[S3]

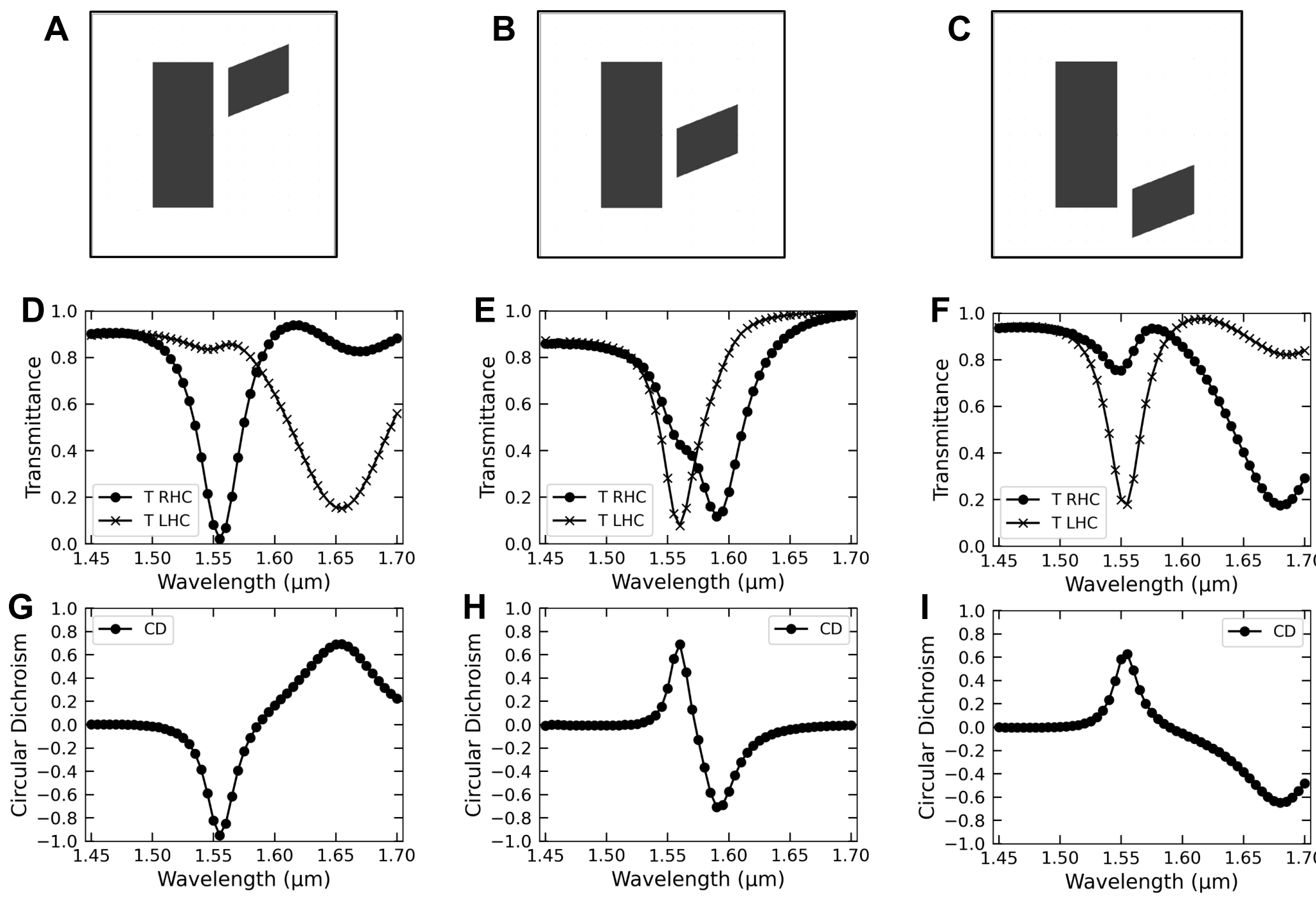

Figure S5: Metasurface made of oblique block unit with 50 nm gap. (A)–(C) Unitcells with different positions of the tilted small block. Gray denotes Si. (D)–(F) Circularly polarized T spectra, corresponding to (A)–(C), respectively. RHC and LHC are equivalent to RCP and LCP, respectively. (G)–(I) CD spectra, corresponding to (A)–(C), respectively.

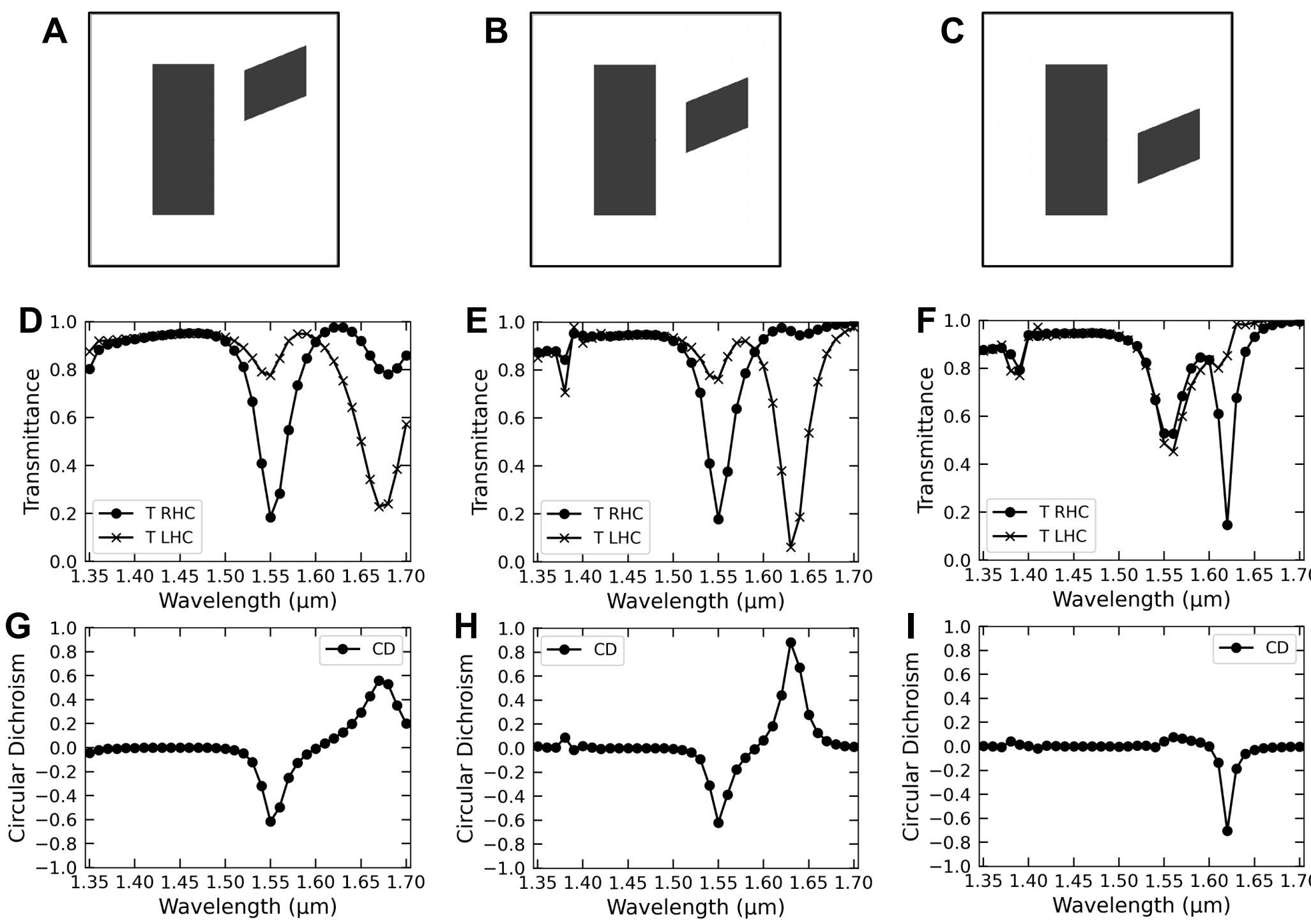

Figure S6: Metasurface made of oblique block unit with 100 nm gap. (A)–(C) Unitcells with different positions of the tilted small block. Gray denotes Si. (D)–(F) Circularly polarized T spectra, corresponding to (A)–(C), respectively. RHC and LHC are equivalent to RCP and LCP, respectively. (G)–(I) CD spectra, corresponding to (A)–(C), respectively.

S6. T and CD Spectra with/without Substrate

To explicitly examine the effect by the substrate on the CD, we computed T and CD spectra in configurations with/without substrates. In Figure S7–S10, T spectra under LCP and RCP are commonly shown with green and orange curves, respectively.

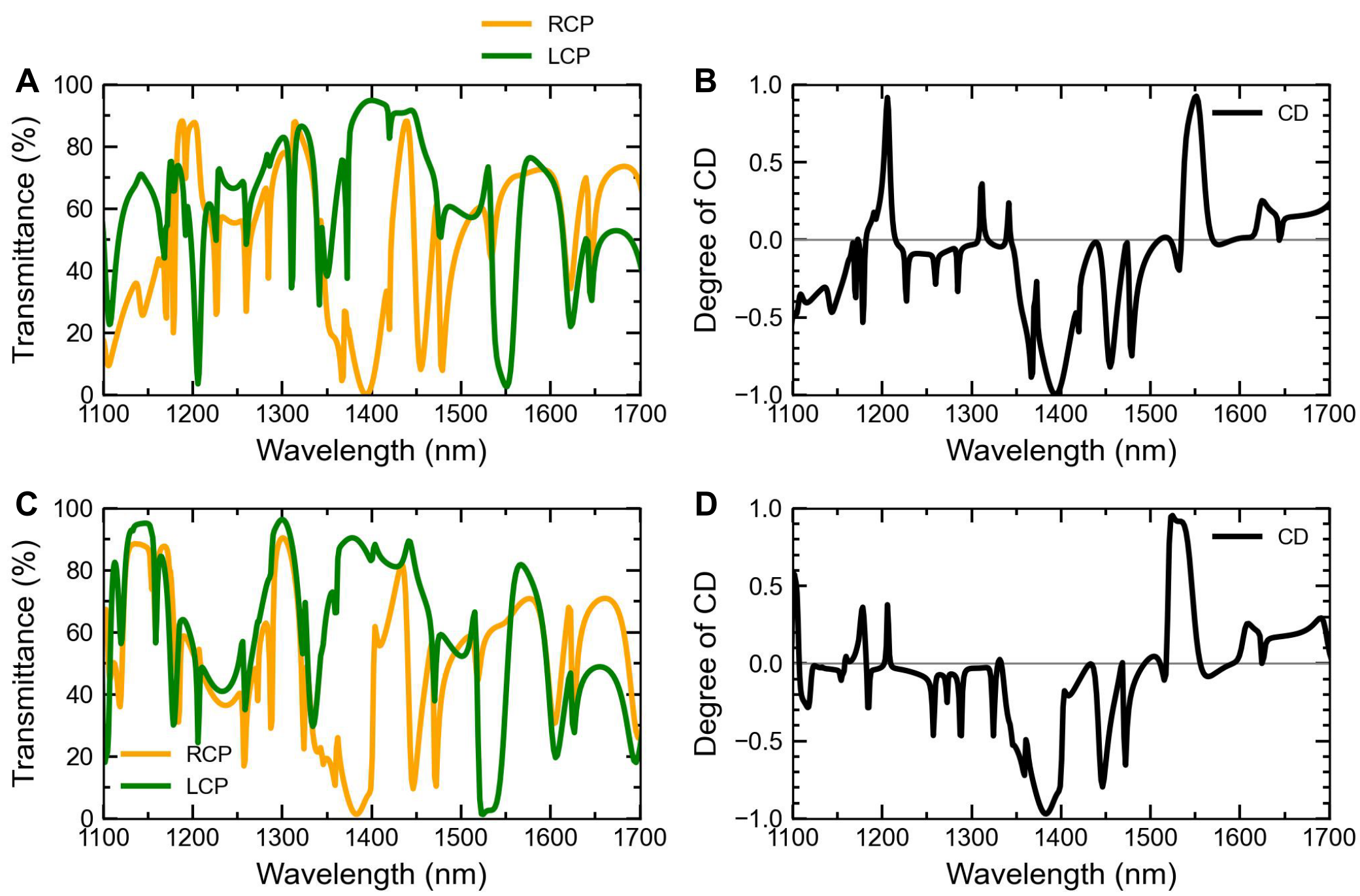

Figure S7: Simulated T and CD spectra of the crossbar-type metasurface. (A, B) T and CD spectra of the metasurface on quartz substrate, respectively. (C, D) T and CD spectra of the metasurface without the substrate, respectively.

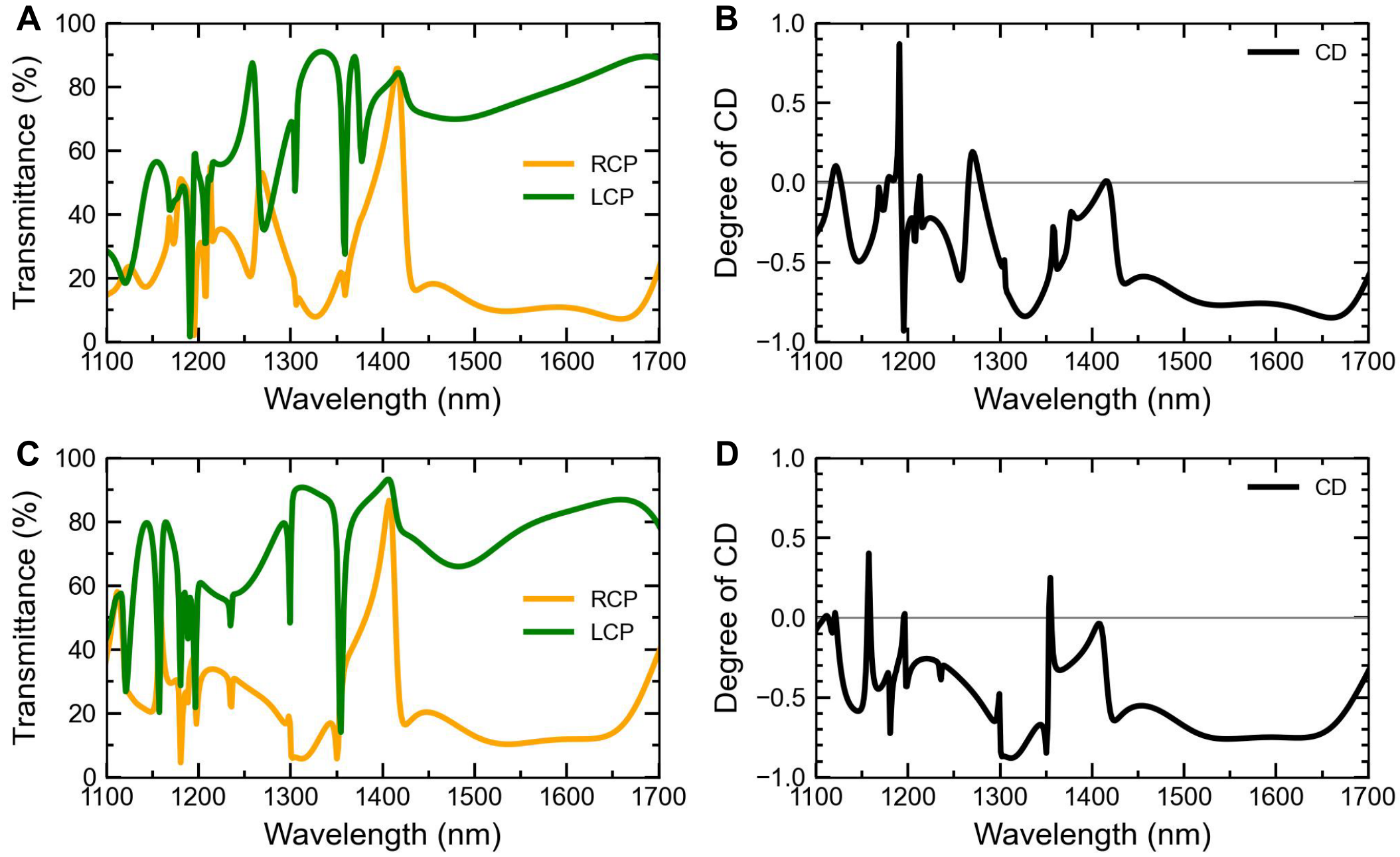

Figure S8: Simulated T and CD spectra of the elliptical-pair metasurface. (A, B) T and CD spectra of the metasurface on quartz substrate, respectively. (C, D) T and CD spectra of the metasurface without the substrate, respectively.

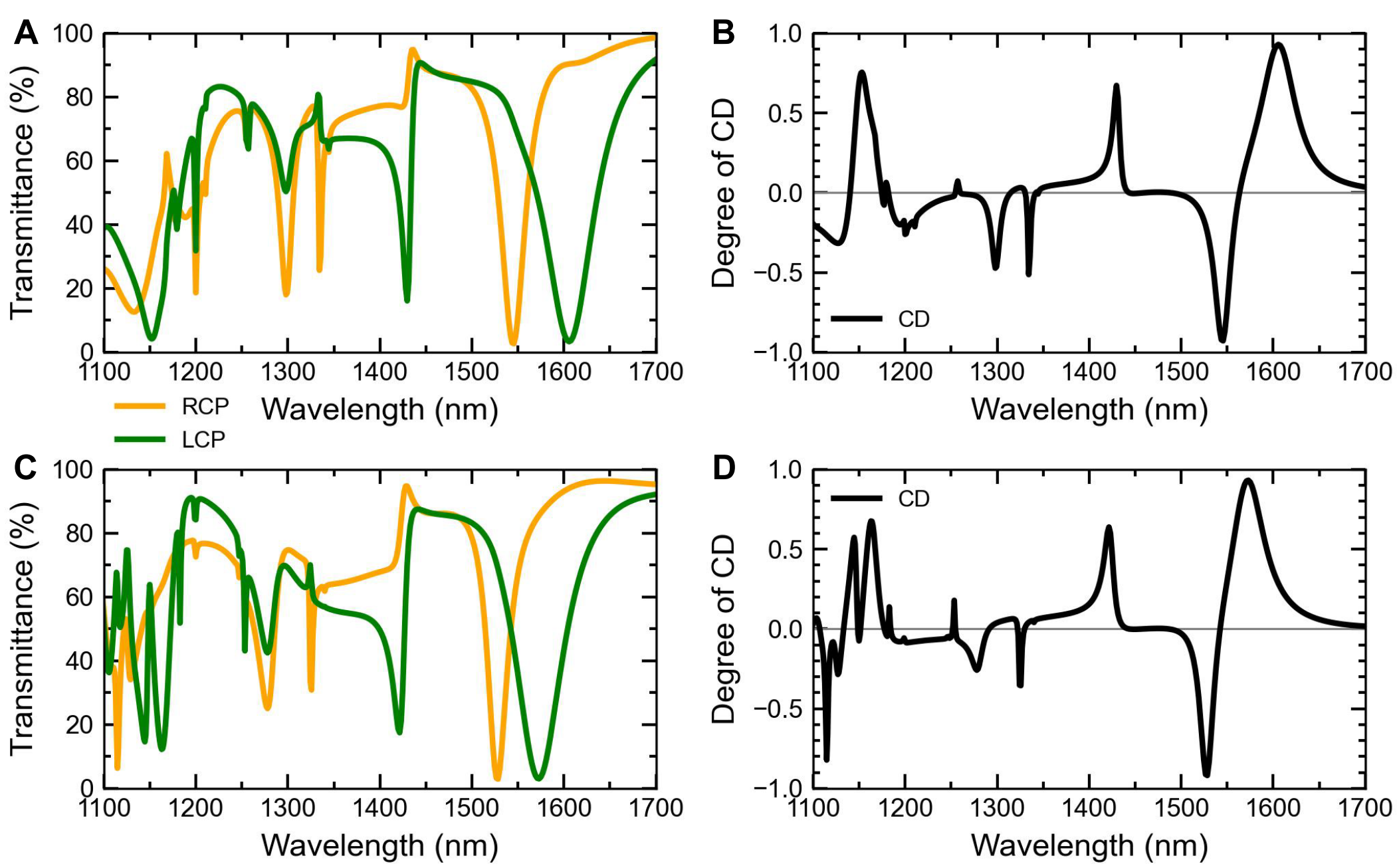

Figure S9: Simulated T and CD spectra of the oblique-block metasurface. (A, B) T and CD spectra of the metasurface on quartz substrate, respectively. (C, D) T and CD spectra of the metasurface without the substrate, respectively. Legend for the T spectra are shown in common with (A) and (C).

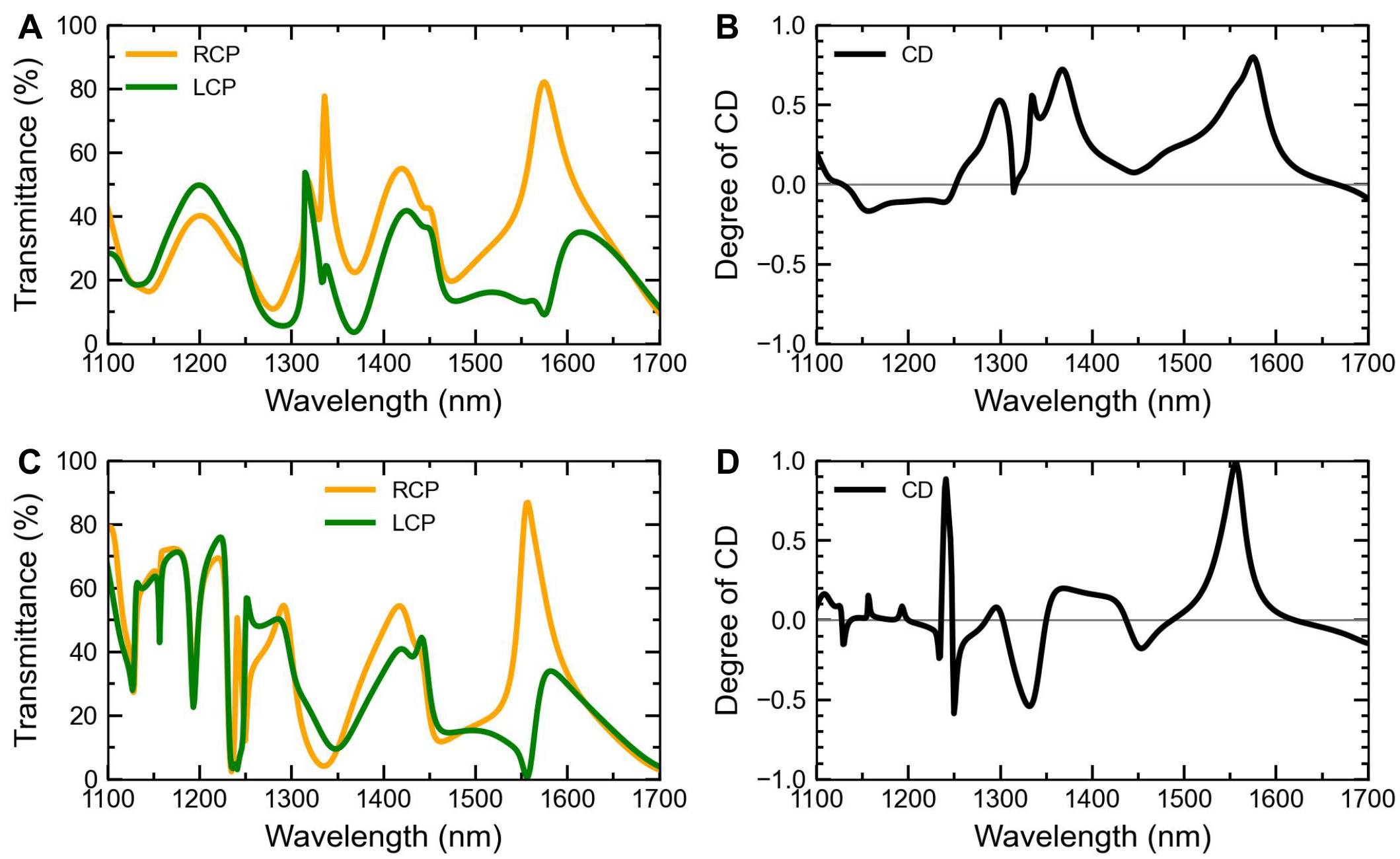

Figure S10: Simulated T and CD spectra of the L-shape metasurface. (A, B) T and CD spectra of the metasurface on quartz substrate, respectively. (C, D) T and CD spectra of the metasurface without the substrate, respectively.

S7. Poynting Field in The L-shape Metasurface

In Figure S11, 3D-view unitcell of the L-shape metasurface (Figure 1) and S_z components in representative xy sections under circular polarizations are shown. Each z positions in Figure S11B–G are indicated by dashed lines in Figure S11A. In this computation, the SOQ layer was set to 500 nm in thickness.

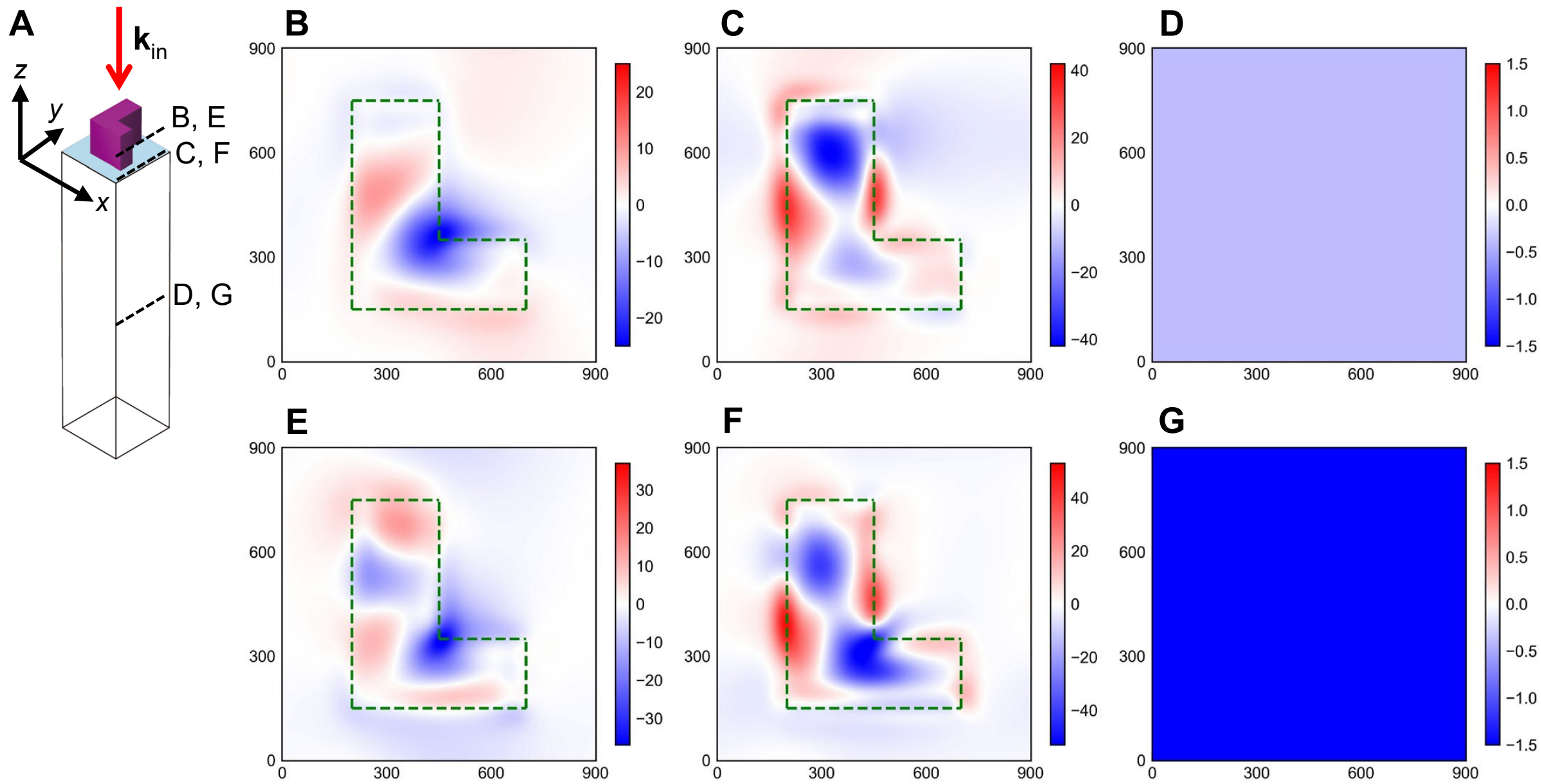

Figure S11: (A) 3D view of unitcell of L-shape structure. (B)–(D) S_z components under LCP $\lambda = 1575$ nm, shown in xy sections at $z = -250$, -500, and -2500 nm, respectively. (E)–(G) S_z components under RCP, shown similarly to (B)–(D), respectively. The z positions in (B)–(G) are indicated by dashed lines in (A).

References

(S1) Li, L. Formulation and comparison of two recursive matrix algorithms for modeling layered diffraction gratings. *J. Opt. Soc. Am. A* **1996**, *13*, 1024–1035.

(S2) Palik, E. D. *Handbook of Optical Constants of Solids II*; Academic: San Diego, CA, USA, 1991.

(S3) Oskooi, A. F.; Roundy, D.; Ibanescu, M.; Bermel, P.; Joannopoulos, J. D.; Johnson, S. G. Meep: A flexible free-software package for electromagnetic simulations by the FDTD method. *Computer Phys. Commun.* **2010**, *181*, 687–702.
